# Dual-Wavelength (UV and Blue) Controlled Photopolymerization Confinement for 3D-Printing: Modeling and Analysis of Measurements

**DOI:** 10.3390/polym11111819

**Published:** 2019-11-06

**Authors:** Jui-Teng Lin, Da-Chuan Cheng, Kuo-Ti Chen, Hsia-Wei Liu

**Affiliations:** 1 New Vision, Inc., New Taipei City 242, Taiwan; jtlin55@gmail.com; 2 Department of Biomedical Imaging and Radiological Science, China Medical University, Taichung City 404, Taiwan; dccheng@mail.cmu.edu.tw; 3 Graduate Institute of Applied Science and Engineering, Fu Jen Catholic University, New Taipei City 242, Taiwan; tony022199@msn.com; 4 Department of Life Science, Fu Jen Catholic University, New Taipei City 242, Taiwan

**Keywords:** kinetic model, dual-wavelength, photopolymerization, spatial confirmation, additive manufacturing, 3D printing

## Abstract

The kinetics and modeling of dual-wavelength (UV and blue) controlled photopolymerization confinement (PC) are presented and measured data are analyzed by analytic formulas and numerical data. The UV-light initiated inhibition effect is strongly monomer-dependent due to different C=C bond rate constants and conversion efficacies. Without the UV-light, for a given blue-light intensity, higher initiator concentration (C_10_) and rate constant (k’) lead to higher conversion, as also predicted by analytic formulas, in which the total conversion rate (*R*_T_) is an increasing function of C_1_ and k’R, which is proportional to k’[gB_1_C_1_]^0.5^. However, the coupling factor B_1_ plays a different role that higher B_1_ leads to higher conversion only in the transient regime; whereas higher B_1_ leads to lower steady-state conversion. For a fixed initiator concentration C_10_, higher inhibitor concentration (*C*_20_) leads to lower conversion due to a stronger inhibition effect. However, same conversion reduction was found for the same H-factor defined by *H*_0_ = [*b*_1_*C*_10_ − *b*_2_*C*_20_]. Conversion of blue-only are much higher than that of UV-only and UV-blue combined, in which high *C*_20_ results a strong reduction of blue-only-conversion, such that the UV-light serves as the turn-off (trigger) mechanism for the purpose of spatial confirmation within the overlap area of UV and blue light. For example, UV-light controlled methacrylate conversion of a glycidyl dimethacrylate resin is formulated with a tertiary amine co-initiator, and butyl nitrite. The system is subject to a continuous exposure of a blue light, but an on-off exposure of a UV-light. Finally, we developed a theoretical new finding for the criterion of a good material/candidate governed by a double ratio of light-intensity and concentration, [*I*_20_*C*_20_]/[*I*_10_*C*_10_].

## 1. Introduction

Polymers for 3D printing and customized additive manufacturing (AM) using various materials such as thermoplastics, polymeric resins and inorganic powders, and under various methods of material extrusion, powder bed fusion and binder jetting have been developed [1,2,3,4,5,6,7,8]. Conventional photolithographic rapid prototyping achieves reaction confinement in depth through patterned irradiation of a resin having a strong absorbance at a specific wavelength and limited to a very thin layer of photo-cured material. Most contemporary stereo lithographic devices use a single wavelength light to initiate polymerization patterned in a plane. However, the single-wavelength irradiation suffers the loss of polymerization confinement by accumulation of non-target light exposure [2,3,4,5,6]. To overcome this drawback, two-color (UV and blue lights), direct-write photolithography was reported, in which the UV-light selectively results polymerization inhibition, concurrent with the blue-light photo-orthogonal, patterned irradiation employed to induce photopolymerization [9,10,11].

Idea dual-wavelength AM systems require the following conditions: (i) the photoinhibition light (UV) must significantly yield enough to cease polymerization, while keeping sufficiently high polymerization rates produced by the initiation light (blue or red); (ii) a wide range of compatible monomers and co-initiators; (iii) the photoinhibition of free-radical chain-growth can be rapidly switched on and off by cycling the UV light; (iv) the formulated resins can be spatially confined using concurrent blue and UV irradiation; (v) rapid elimination of the inhibitor species in the dark, or after cessation of UV exposure; (vi) large polymerization inhibition depth adjacent to the projection window and (vii) continuous part production at translation speeds of several hundred millimeters per hour. Above idea conditions allow for rapid, single-exposure fabrication of complex structures, which cannot be easily achieved by conventional single-wavelength methods.

Previously reported inhibition layers via oxygen inhibition are typically only tens of micrometers thick and thus it requires the use of low-viscosity resins or fabrication of objects with small cross sections. [6,7]. Single-wavelength, conventional and diffusion-reliant methods with a large inhibition thickness (IT) and high photoinitiation rates offer a continuous and rapid object printing [8,9,10]. However, they suffer the issues of separation resin reflow. In dual-wavelength systems, the IT may be reduced by decreasing irradiation intensity and thus increase the print speeds as polymerization proceeds closer to the projection window. For a given resin composition, the IT depends on the ratio of inhibitor to initiator absorbance, quantum yield of the radicals. IT also depends the reaction rates among the radicals and monomers, initiator, co-initiator and inhibitor and the dual-wavelength light intensity and dose (or exposure time). The advantages of dual-wavelength concurrent inhibition and initiation photopolymerization include: (i) controllable high vertical print speeds, (ii) eliminating the need for thin, oxygen-permeable projection windows, (iii) single-step fabrication of cured materials and (iv) rapid generation of personalized products. One additional advantage is that the reflow into the inhibition volume during printing can be optimized for large cross-sectional area parts.

Dual-wavelength photopolymerization confinement (PC) was reported in both parallel [10] and perpendicular [11] concurrent irradiation schemes. In the parallel scheme, the volumetric PC was achieved by inhibition volume depth controlled by varying the ratio of the intensities of the UV and blue lights, where print speeds of 2 m/h have been achieved in a wide variety of resins including acrylates, methacrylates and vinyl ethers. In addition, by varying the intensity of the light source on a per-pixel basis, the system can perform surface topographical patterning in a single exposure/layer with no stage translation [10]. In the perpendicular scheme, photopolymerizations were reported to confine in depth the region polymerized resin, in which two perpendicular blue and UV lights, independently effect polymerization initiation and inhibition, respectively [11].

As reported by van der Laan et al. [11], the effectiveness of a photoinhibitor is strongly monomer-dependent, which also requires: (i) a high conversion of blue-photoinitiation in the absence of the UV-active inhibitor; (ii) a strong chain termination with significant reduction of blue and UV conversion in the presence of UV-active inhibitor and (iii) short induction time or rapid elimination of the inhibitor species in the dark (or absence of UV-light), such that the initiation–inhibition cycles may be switched on and off rapidly. Fast switching-time may be achieved by high conversion rate, or high blue-light intensity, the triple-state quantum yield, and the absorption constant of the monomer resin. However, we note that higher light intensity achieves a faster photoinitiation, but suffers a lower saturation value, or lower conversion. Moreover, a short induction time may be achieved by high UV intensity or large inhibitor-concentration, and it also requires minimum impurity and oxygen, which cause a delayed curing of the resin.

The monomer-dependence of a dual-wavelength PC was reported by van der Laan et al. [11], in which different monomers have different C=C bond rate constants (K) under the exposure of blue, UV and blue + UV. For example, bisphenol ethoxylate diacrylate (BPAEDA) resins formulated with camphorquinone (CQ) and ethyl 4-(dimethylamino)benzoate (EDAB) have a maximum conversion rate constant, for 0% butyl nitrite (BN), Kmax = 0.675 (at blue + UV), which reduces to 0.0106 (for 1%BN), a factor of 64 reduction. Therefore, it is a better candidate than trimethylolpropane triacrylate (TMPTA), which only has a three times reduction of Kmax.

Although the mechanism for radical-mediated polymerization initiation and inhibition in dual-wavelength system and the simple formulas for the associated printing speed and inhibition volume thickness [11] were reported, however, no detailed kinetics or the conversion efficacy have been theoretically reported in a dual-wavelength, thick polymer system. We have previously reported the kinetics and modeling of a single-wavelength radical-mediated photopolymerization in single-initiator [12,13,14], two-initiator [15] and two-component system [16,17]. This study will extend our previous modeling to a 2-wavelength, 3-initiator system. We will focus on the following issues: the roles of the concentration of the blue-light active initiator and its co-initiator, and the UV-light-active inhibitor (BN); the role of reaction rates and light intensity on the conversion profiles; the polymerization inhibition depth and printing speed and induction time, which is desired for a fast on-off switching control of PC. Finally, analytic formulas and numerical results will be utilized to analyze the measured data of de Beer et al. [10] and van der Laan et al. [11].

## 2. Materials and Methods

### 2.1. Photochemical Kinetic

**Figure 1:**
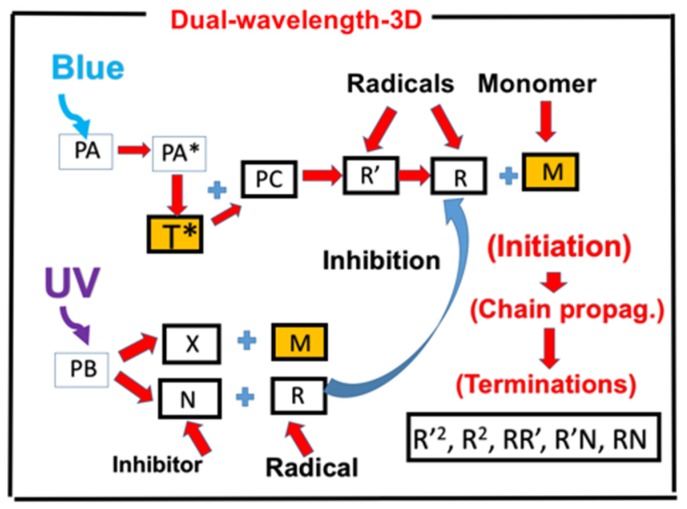
Schematics of photochemical pathways of dual wavelength photopolymerization; in which crosslinkers are formed via two pathways, via the photoinitiator PA (under a blue light), and PB (under a UV light). The initiation radicals R and [X] crosslink with the monomer [M]; whereas the inhibition radicals [N] reduces the conversion efficacy by reducing the active radicals (R’ and R). Shown also is the co-initiator (PC), which reacts with the triplet state of PA (T*) forming an intermediate radical (R’). Bimolecular termination of R’ produces a propagating radical (R) which leads to crosslinks; terminations could be also resulted by the interaction of R and R’, and R and [N].

As shown by Figure 1, a dual wavelength (UV and blue light) radical-mediated system consists of a photoinitiator, PA, a co-initiator, PC and another photosensitizer, PB, which serves as a photoinhibitor for the monomer conversion. The blue-light (at 470 nm) excites photoinitiator (PA) producing excited states, PA* and triplet state T*; whereas the UV-light (at 365 nm) photodecomposes the photoinhibitor (PB) to produce an inhibition radical [N], and an initiation radical [X], which could interact with the monomer [M] for crosslinking. The PA triplet state (T*) could interact with the co-initiator, PC, forming the primary radical (R’), which reacts with PA for a chain propagation and produces more radical (R). Bimolecular termination of R’ produces the propagating radical (R), which leads to crosslink; terminations may be also resulted by the recombination of R, interaction of R’ and R, [N] and R’ and R.

Example of the above described dual-wavelength system were reported by de Beer et al. [10] and van der Laan et al. [11], in which methacrylate conversion under the exposure of blue (470 nm) and UV (365 nm) light was measured for the photopolymerization of a methacrylate resin formulated with camphorquinone (CQ, as PA), ethyl 4-(dimethylamino)benzoate (EDAB, as a co-initiator, PC) and butyl nitrite (BN), as the UV-activated initiator, PB). The photochemical decomposition of butyl nitrite (BN) results in nitric oxide (N), an efficient inhibitor and alkoxide radical (X) for extra polymerization initiation, besides the reactive radical (R).

Short-hand notations for the concentration of various components were used: C_1_, C_2_ and C_3_ for the ground state concentration of PA, PB and PC, respectively and [M] for the monomer, the kinetic equations for the dual-color and 5 radicals (R’, R, [N] and [X]) system are derived as follows [12,15,16]:

(1) $$\frac{\partial C_{1}}{\partial t}=-{\mathrm{gB}}_{1}C_{3}C_{1}$$

(2) $$\frac{\partial C_{2}}{\partial t}=-{g\prime B}_{2}\left[M\right]C_{2}$$

(3) $$\frac{\partial C_{3}}{\partial t}=-{\mathrm{gB}}_{1}C_{3}C_{1}+2k_{T}R^{2}+k_{22}R\left[N\right]$$

(4) $$\frac{\partial \left[N\right]}{\partial t}=B_{2}C_{2}-k_{22}R\left[N\right]$$

(5) $$\frac{\partial \left[X\right]}{\partial t}=B_{2}C_{2}-(k_{6}+k_{8}\left[M\right])\left[X\right]$$

(6) $$\frac{\partial R\prime }{\partial t}={\mathrm{gB}}_{1}C_{1}C_{3}-(2k_{T}R\prime +k_{12}R)R\prime$$

(7) $$\frac{\partial R}{\partial t}=-2k_{T}R^{2}-\left(k^{\prime }\left[M\right]+k_{12}R\prime +k_{22}\left[N\right]\right)R+2k_{T}{R\prime }^{2}$$

(8) $$\frac{\partial \left[M\right]}{\partial t}=-(k_{8}\left[X\right]+k\prime R)\left[M\right]$$

where B_1_ = b_1_I_1_(z,t), B_2_ = b_2_I_2_(z,t)I_3_(z,t)/I_30_, B_3_ = (b_3_/b_2_)B_2_, g =1/(k_57_ + kC_3_), g’ = 1/(k_68_ + [M]), k_57_ = (k_5_/k_7_), k_68_ = (k_6_/k_8_) and k = (k_3_/k_7_). R_E_ is the C_1_ regeneration term given by R_E_ = k_22_[N]R + 2k_T_R^2^. b_j_ = 83.6a_j_q_j_w_j_; a_j_ is the extinction coefficient for PA, PB and PC (with *j* = 1,2,3); the light wavelength (in cm), w_1_ for the blue (at 470 nm) and w_2_ for UV (at 365 nm) and light intensity I_j_ (z,t) in mW/cm^2^; q_j_ is the quantum yields of the PA triplet state and PB radical.

Equation (6) gives the generation of radical R’ via the interaction of T* and the co-initiator (PC), given by k_3_T*C_3_, with the steady-state T* = (g/k_7_)B_1_C_1_. The UV light intensity, I_2_(z,t), is absorbed mainly by PB, in which the UV conversion of PA monomer is reduced by the presence of PB. On the other hand, the blue-conversion of PA could be significantly reduced by the UV-generated radical of PB, such that the inhibition depth may be controlled by the on-off of UV light (more will be discussed later). All the reaction rate constants are defined by the associated coupling terms. For examples, in Equation (7), k’ is for the reaction of monomer and radical R, which has a relaxation rate k_5_; k_12_ is for the radical interaction of R’ and R and both have a bimolecular termination rate of k_T_. More detail derivation and definition of rate constants in g and g’ have been previously published [12,15].

Using the quasi-steady-state conditions of [12,15] d[N]/dt = d[X]/dt = d[R’]/dt = 0, we obtain steady-state radicals given by [N] = B_2_C_2_/(k_22_R), [X] = g’B_2_C_2_/k_8_, and Equation (8) becomes:

(9) $$\frac{\partial \left[M\right]}{\partial t}=-R_{T}\left[M\right]$$

(10) $$R_{T}=g{\prime B}_{2}C_{2}+k\prime R$$

where R_T_ is a total rate constant, which consists of two crosslink components attributed from the interaction of the monomer and [X] and R, respectively. Furthermore, the steady-state radicals, R’ and R, are given by:

(11) $$2k_{T}{R\prime }^{2}+k_{12}R\prime -{\mathrm{gB}}_{1}C_{1}C_{3}=0$$

(12) $$2k_{T}R^{2}+\left(k^{\prime }\left[M\right]+k_{12}R\prime \right)R-(2k_{T}{R\prime }^{2}-B_{2}C_{2})=0$$

Solving for Equations (11) and (12), we obtain:

(13) $$R=\left(\frac{1}{4k_{T}}\right)\left[-G+\sqrt{G^{2}+8k_{T}H}\right]$$

(14) $$H={\mathrm{gB}}_{1}C_{1}C_{3}-B_{2}C_{2}$$

where G = k’[M] + 2k_12_R’. Equation (13) may be further approximated to R = (0.5H/k_T_)^0.5^ − k’[M](1 − d), with d = 0.5G^2^/(8k_T_H), for 2k_12_R’ << k’[M], which shows that R and efficacy, are increasing the function of H. The balance point of inhibition depth is defined by when R = 0, or 8k_T_H = 0 or gB_1_C_1_C_3_ = B_2_C_2_, in which the PA initiated radical (R) is completely inhibited/consumed by the PB’s radical, [N]. We will have more discussion later.

The dynamic light intensity of blue (I_1_) and UV (I_2_) are given by, when they are applied to the resin orthogonally and separately [13,15]:

(15) $$I_{j}\left(z,t\right)=I_{j0}\exp\left[-A_{j}z\right]$$

(16) $$A_{j}\left(z,t\right)=2.3\left(a_{j}C_{j0}+Q_{j}\right)-A_{j1}t$$

where a_j_ is the extinction coefficients of PA (for j = 1) and PB (for j = 2) and their photolysis products, respectively; Q_j_ is the absorption coefficient of the monomer at the blue and UV wavelength. Most previous modeling [8,9,10,11,12,13] assumed a constant C (z, t) in Equation (2.b). Our analytic formulas in this article will use a time-average of A (z, t) to count for the dynamic of light intensity due to PA and PB depletions. Accurate solutions of Equations (1) and (8) require numerical simulations (to be shown later). For analytic formulas, we will use approximated analytic formulas for the light intensity and the PI and PE concentration. The expressive closed forms of I_j_(z,t) and C_j_(z,t) allow us to solve for the first-order and second-order solutions of R, [M] and the conversion efficacy.

### 2.2. Analytic Formulas for Efficacy

The monomer conversion efficacy for a bimolecular termination process is given by C_EFF_ = 1 − [M]/[M]_0_ = 1 − exp(−S), with [M]_0_ being the initial monomer concentration, and the S-function is given by the time integral of the total rate factor R_T_ given by Equation (1.f), d[M]dt = –R_T_[M], in which R_T_ has three components defined by the coupling of the monomer [M] and the triplet-state, PA-radical and PB radical, respectively.

For k_53_ << kC_3,_ k_68_ << k”[M], gC_3_ = g’[M] = 1, the solutions of Equations (1) to (3) are available by the approximated analytic formulas for I_j_ (z,t) and C_j_ (z,t), with j = 1,2,3, for PA, PB and PC, as follows [12,13]:

(17) $$I_{j}\left(z,t\right)=I_{j0}\exp\left[-A_{j}z\right]$$

(18) $$C_{j}\left(z,t\right)=C_{j0}\exp\left[-B\prime_{j}t\right]$$

(19) $$A_{j}\left(z,t\right)=2.3\left(a_{j}C_{j0}+Q_{j}\right)-A_{j1}t$$

where B’_j_ = b_j_I_j0_exp(−A_j_’z), A_j1_ = 2.3(a_j_ − b’_j_)C_j0_I_j0_b_j_z_,_ with A_j_’ is the time-averaged absorption given by Aj’ = 1.15(a_j_ + b’_j_) + 2.3Q_j_, bj’ is the extinction coefficient of the photolysis products. We note that the –A_j1_t term represents the decrease of A_j_’, or increase of light intensity due to concentration depletions of PA, PB and PC.

Using Equations (17) and (18) for the total rate faction, R_T_, we solved Equation (9) to obtain the total efficacy given by CEFF = 1−[M]/[M]_0_ = 1−exp(−S), where S is the time integral of R_T_, which requires a numerical integration, in general. For analytic solutions, two cases were considered. For gB_j_C_j_ << k’R, case (i) H >> G, k’R = KH^0.5^, with K = 0.5k’/k_T_^0.5^; case (ii) H << G, k’R = k’H/G; where H^0.5^ may be further reduced to H^0.5^= (B_1_C_1_)^0.5^ – 0.5(B_2_C_2_)/(B_1_C_1_)^0.5^, for (B_2_C_2_) << (B_1_C_1_) and gC_3_ = 1, for k = k_3_/k_7_ = 1.

For case (i) H >> G, C_EFF_ = 1 − [M]/[M]_0_ = 1−exp(−S), with S-function is given by:

(20) $$S=K\sqrt{D_{10}}[E_{11}-0.5(D_{20}/D_{10})E_{12}]$$

(21) $$E_{11}=[1-\exp(-G_{31}t)]/G_{31}$$

(22) $$E_{12}=[1-\exp[-(G_{32}-G_{31})t]/(G_{32}-G_{31})$$

where initial values: D_j0_ = B_j0_X_j_,G_3j_ = 0.5(B_j0_ − A’_1j_), with B_j0_ = b_j_I_j0_C_j0_, X_j_ = exp(−A’_j_z)_,_ A’_j_ = 1.15(a_j_ + $b_{j}$)C_j0_ + 2.3Q_j_, is a mean value of A_j_(z,t). We note that Equation (20) reduces to our previous formula for one-wavelength system with B_20_ = 0 and H = B_1_C_1_.

For case (ii), H << G, and for k’[M] << k’R, k’R = KH/[M], with K = 0.5k’/k_T_^0.5^, Equation (9) becomes:

(23) $$\frac{\partial \left[M\right]}{\partial t}=-\mathrm{KH}\left(t\right)$$

Therefore, the efficacy is given by the time integral of H(t)/[M]_0,_ or:

(24) $$C_{EFF}=KS/{\left[M\right]}_{0}$$

with S given by Equation (20). The steady-state of Equations (21) and (22) are given by when E_11_ = 1/G_31_, E_12_ = 1/(G_32_ − G_31_), whereas transient state is given by E_11_ = E_12_ = t. Therefore, the inhibition effect given by the second term of Equation (21) is proportional to B_20_/(B_10_)^0.5^/(G_32_ − G_31_), with B_j0_ = b_j_I_j0_C_j0_, for steady-state; and [tB_20_/(B_10_)^0.5^] for transient state. Numerical data will be shown later. We also note that for a given B_1_C_1_, the radical R is a decreasing function of the ratio of R_AB_ = (B_2_C_2_)/(B_1_C_1_)^0.5^. Therefore, the same R_AB_ reaches the same efficacy. This feature will be numerically shown later.

### 2.3. The Inhibition Depth and Time

Polymerization inhibition depth adjacent to the projection window is a critical parameter for continuous stereolithographic fabrication. One may find the inhibition depth (z_H_) defined by the balance point of initiation and inhibition rate, or when R = 0, or H = 0. We find from Equation (14),

(25) $$z_{H}\left(t\right)=\frac{1}{A_{2}+A_{3}-A_{1}}\ln\left(\frac{B_{20}C_{2}}{gB_{10}C_{1}C_{3}}\right)$$

where B_j0_ = b_j_I_j0_, and C_j_ (t) are the z-averaged function of C_j_ (z,t). We note that Equation (8) defines an inhibition coefficient defined by β = (b_2_/b_1_)[C_2_/(gC_1_C_3_)], which depends on a multifactor and rate constants related by g = 1/(k_57_ + kC_3_). Our formula is more general than that of de Beer et al. [10], which is our special case when A_3_ = 0, and C_2_ = gC_1_C_3_, such that Equation (25) reduces to Equation (1) of de Beer et al. [10]: z_H_ = (1/(A_2_ – A_1_)ln[βI_20/_/I_10_], with β(k37 + kC3 = [M])b_2_/b_1_. We note that b_j_ = 83.6a_j_q_j_w_j_, which is defined by the extinction coefficient _for_ PA, PB and PC (with j = 1,2,3); the light wavelength, w_1_ for the blue (at 470 nm) and w_2_ for UV (at 365 nm) and the quantum yields (q_j_). Moreover, in our more general formula, β is also proportional to 1/g = k_57_ + kC_3_, defined by the rate constants of k_57_ and k = k_3_/_7_.

Due to the time-dependence of C_j_(z,t), Equation (25) in general is time-dependent, which was assumed as time-independent by de Beer et al. [10], when C_j_ reaches a steady-state or remains as its initial value, in which the initiators depletion is ignored. To explore this dynamic feature, one may define an inhibition time (T_H_) given by when the radical R = H = 0. For a common situation that that C_1_(t) = C_10_ exp(-B’t), with PA has a depletion rate, B’, much larger than that of PB and PC, such that C_2_(t) = C_20_, C_3_(t) = C_30_, both are much slowing decay function of time (to be shown by our numerical data later), we obtained an analytic formula:

(26) $$T_{H}=\frac{1}{B\prime }\ln\left(\frac{I_{10}}{\beta^{\prime }I_{20}}\exp\left[\left(A_{2}+A_{3}-A_{1}\right)z\right]\right)$$

where β′= (b_2_/b_1_)[C_20_/(gC_10_C_30_)]. Equation (26) shows that T_H_ is an increasing function of the depth (z), but a decreasing function of the concentration ratio C_20_/(C_10_C_30_), i.e., higher inhibitor concentration C_20_, results to a shorter inhibition time, which is desired for a faster on-off switching mechanism.

The thickness of this polymerization inhibition volume adjacent to the projection window is a critical parameter for continuous stereolithographic fabrication. A minimum intensity ratio of UV and blue light, Rmin = (I_20_/I_10_)_crit_ defined by which initiation and inhibition rates are balanced to generate an inhibition depth, z_H_ = 0 in Equation (25), and can be calculated by when Rmin = 1/β′ = gC_10_C_30_/[(b_2_/b_1_)C_20_], which is dependent on resin composition ratios and rate constants. de Beer et al. [10] reported β′=1 in a TMPTA-based system.

As the above formulas, Equation (8) is based on H = 0 as also defined by de Beer et al. [10]. A more accurate definition should be based on the time integral of R_T_, and defined by the S-function higher than a critical value, S > S_T_, or efficacy C_EFF_ > C_T_, where S_T_ = ln [1/(1 – C_T_)], which can only be calculated numerically (to be shown later).

### 2.4. Print Speed

Based on Equation (13), the maximum print speed (Smax) as defined by de Beer et al. [10], when the dose difference of blue light and UV light equals to a critical value (E*), and B_1_ = βB_2_, we obtain a similar formula:

(27) $$S_{\max}=\frac{B_{20}-{\beta B}_{10}}{E^{*}}$$

which, however, has a more complex function of β = (C_2_b_20_/b_10_)/(gC_1_C_3_), than the simplified function of de Beer et al. [10], with β = _20_/b_10_. A more accurate definition would be based on the S function, or time integral of Equation (10), rather than light dose given by Equation (20). However, S_max_ needs numerical result integral of Equation (10), which is to be shown later.

### 2.5. Curing Depth

There are two ways to define the curing depth of the green light: the simple one is defined by when the blue light dose, I_10_t, is larger than a threshold value of E_TH_. Using the time integral of Equation (17) with neglected A_1_t, we obtain,

(28) $$z_{C}=\frac{1}{A_{1}}\ln\left(\frac{I_{10}t}{E_{\mathrm{TH}}}\right)$$

The above formula is the same as that of de Beer et al. [10]. However, the more accurate definition of curing depth is given by when the blue-light conversion efficacy, given by Equation (20), is higher than a critical value, C_EFF_ > C_T_, or when S > S_T_, with S_T_ = ln [1/(1 − C_T_)]. We obtain:

(29) $$T_{C}=\left(\frac{2}{B\prime }\right)\ln\left[B\prime /\left(K\sqrt{0,5X\prime B}\right)-1\right]$$

where B’ = (b_1_I_10_C_10_), and X’ = exp(−A’z_C_), with A’ = 1.15(a’_1_ + b’_2_)C_10_ + 2.3Q_1_, is a mean value of A_1_(z,t).

## 3. Results and Discussion

Numerical results based on Equations (1) to (8) and the steady-state radical given by Equation (13), and using the light intensities given by Equation (17) are shown as follows. We first showed the conversion for the case of blue-light only, i.e., when B_2_ = 0 (no UV light) for various concentration of the initiator, C_10_ = (0, 0.5, 1.0, 3.0)%, coupling parameter b_1_, which is given by the absorption coefficient and blue-light intensity and also the role of the crosslink rate constant (k’), which gives the conversion in Equation (10). For simplicity, our modeling was limited to the surface layer of the resin, that is for z = 0. Spatial conversion profiles can be found in our previous study, which is limited to a single-wavelength system [13,14].

We then presented the inhibition effect (IBE) on the conversions under the exposure of both UV and blue light for various inhibitor concentrations. As shown by Equation (9), the conversion efficacy is governed by the H-function of Equation (14), or B_1_C_1_–B_2_C_2,_ as suggested by our analytic formulas, Equation (6), our numerical input will be the initial values of B_j_ or b_j_ (with j = 1,2) rather than the light intensity or the absorption coefficients. We also showed that the IBE was strongly monomer-dependent, as reported by van der Laan et al. [11] by various reaction rate constants (k’, k_T_). The numerically produced temporal profiles were analyzed by our analytic formulas of Equation (20). Finally, our numerical profiles were fit to the measured data of de Beer et al. [10] and van der Laan et al. [11] in an on-off scheme.

### 3.1. Efficacy Temporal Profiles

**Figure 2:**
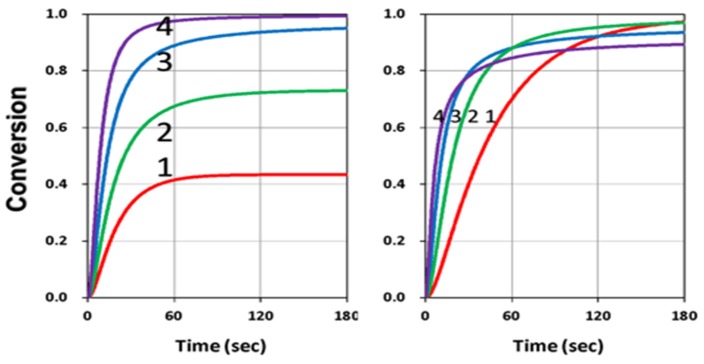
Conversion profiles of blue-light (without UV-light) for (left Figure) C_10_ = (0.05, 0.1, 0.2, 0.4) %, for curve (1,2,3,4), for fixed b_1_ = 0.1; and (right Figure) b_1_ = (0.015, 0.05,0.15,0.5), for fixed C_10_ = 0.2 %; for C_30_ = 0.5 %, [M]_0_ = 0.2 %, k’ = 1.0, k_T_ = 0.5, k_57_ = (k_5_/k_7_) = k_68_ = (k_6_/k_8_) = k” = 35 (1/s).

We first showed the conversion of blue-light only (without the UV-light). Figure 2 (left Figure) shows that higher initiator concentration, C_10_, led to higher conversion, as also predicted by our Equation (10), in which the total conversion rate (R_T_) was an increasing function of k’R, which was proportional to [gB_1_C_1_]^0.5^. However, the coupling factor b_1_ played a different role that higher b_1_ led to higher conversion only in the transient regime; whereas higher b_1_ led to lower steady-state conversion (as shown by right Figure). This unique reverse effect in steady-state was also predicted by our analytic Equation (20).

**Figure 3:**
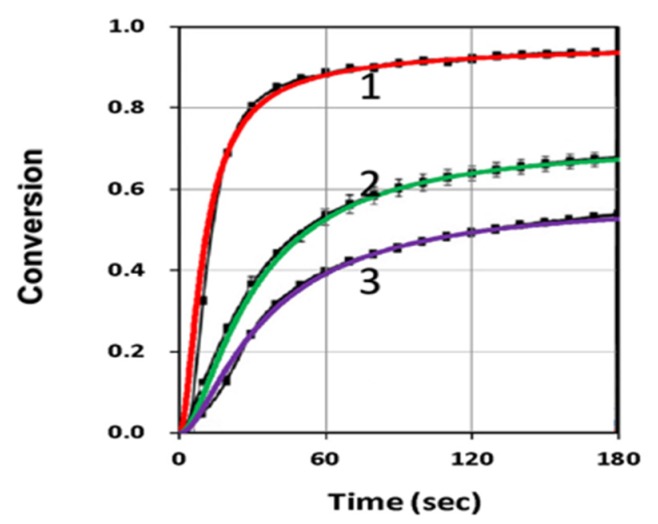
The same as Figure 2 but for various k’ = (1.0, 0.3, 0.19), for curves (1,2,3), for fixed C_10_ = 0.2%, and adjusted b_1_ = (0.15, 0.05, 0.05) to fit the measured data of de Beer et al. [10].

Figure 3 shows that higher coupling rate constant (k’) led to higher conversion, as also predicted by Equation (10). These calculated profiles fit well with the measured data of de Beer et al. [10], for various resin formations (shown by their Figure 3).

**Figure 4:**
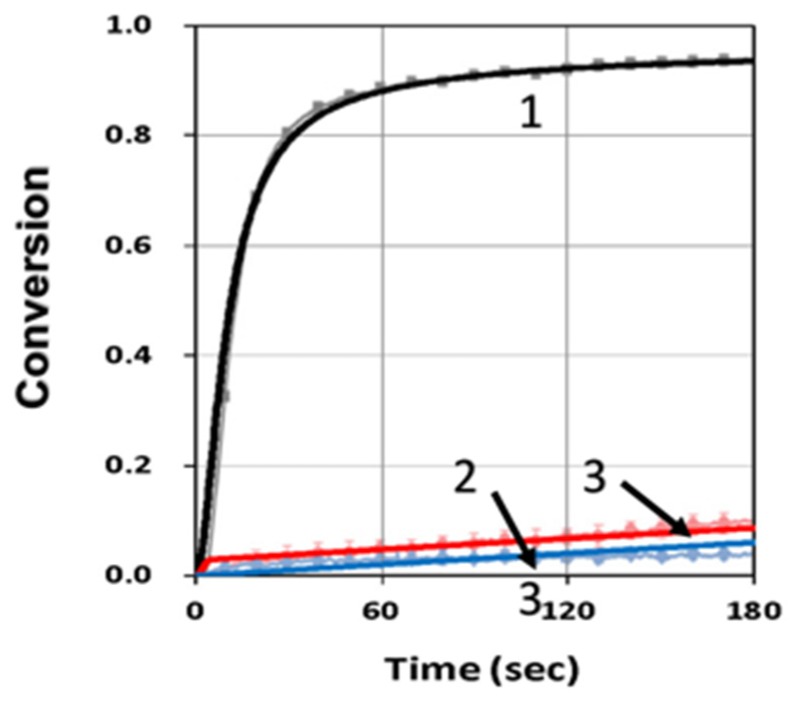
Conversion profiles for blue-only (black curve—1), UV-only (blue curve—2) and both-light (red curve—3), for C_10_ = 0.2%, C_30_ = 3.0%, b_1_ = 0.1, b_2_ = 0.007, k” = 35; where solid color curves are calculated and bars are measured data of de Beer et al. [10].

**Figure 5:**
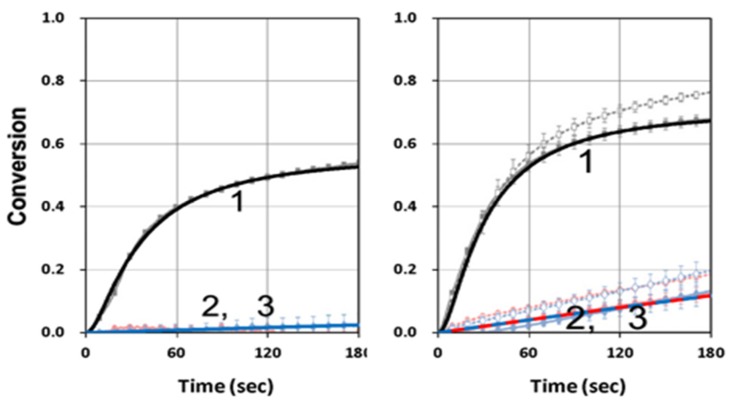
Same as Figure 4, but for different monomers governed by various k” = 60 (left Figure) and k” = 150 (right Figure), for b_1_ = 0.05, b_2_ = 0.007, k’ = 1.0, where bars are measured data of de Beer et al. [10].

Figure 4 shows the conversion profiles for blue-only, UV-only and both-light and compared with measured data of de Beer et al. [10], with fit parameter of k’, k_T_ and b_j_. Conversion of blue-only were much higher than that of UV-only and UV-blue combined, in which a high inhibitor concentration (C_20_) resulted in a strong reduction of blue-only-conversion, such that the UV-light served as the turn-off (trigger) mechanism for the purpose of spatial confirmation within the overlap area of UV and blue light. Figure 5 shows the conversion profiles under the same conditions as that of Figure 4, but for different resin formations, which were specified by our parameter k”. As reported by van der Laan et al. [11], different monomers have different C=C bond rate constants (K) under the exposure of blue, UV and blue + UV. For example, bisphenol ethoxylate diacrylate (BPAEDA) resins formulated with camphorquinone (CQ) and ethyl 4-(dimethylamino)benzoate (EDAB) had a maximum conversion rate constant Kmax= 0.675 (at blue + UV) for 0% butyl nitrite (BN), and reduced to 0.0106 (for 1%BN), a factor of 64 reduction, Therefore, it was a better candidate than trimethylolpropane triacrylate (TMPTA), which only had a three times reduction of Kmax.

Similar to Figure 4, Figure 5 also shows a significant reduction of conversion under the UV-light initiated inhibition of the radical (R). This is one of the key factors to achieve efficient photopolymerization confirmation (PC).

**Figure 6:**
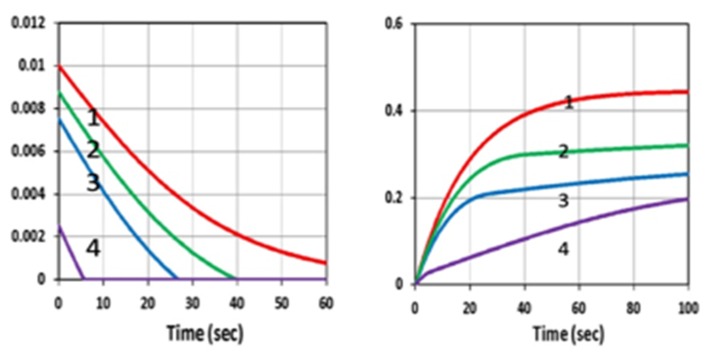
The initiation radical (R, left) and conversion (right) profiles in the presence of UV light; for various inhibitor concentration, C_20_ = (0, 1.0, 2.0, 3.0), for curves (1,2,3,4; red, green, blue, violet), for b_1_ = b_3_ = 0.1 (1/s/%), C_10_ = 0.2 (%), C_30_ = 0.5 (%), [M]_0_ = 0.2 (%); k’ = 1.0 (1/s), k_48_ = 1.0 (1/s), k_37_ = 1.0 (1/s), k_57_ = 0.01 (1/s).

**Figure 7:**
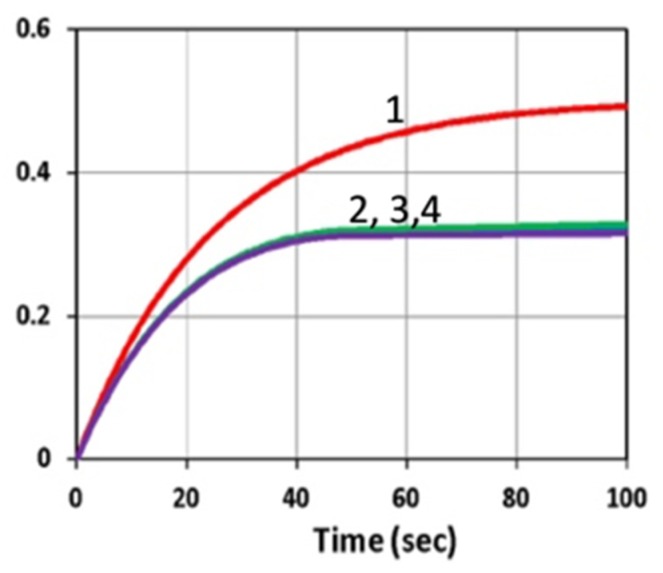
The same as Figure 6, but for a fixed difference of [b_1_C_10_ − b_2_C_20_] = 0.003, for C_20_ = 0 (curve-1) and C_20_ > 0, for curves (2,3,4) showing the overlapping of these three curves.

Figure 6 shows the initiation radical (R, left) and conversion (right) profiles in the presence of UV and blue light, for various inhibitor concentration (C_20_), in which, for a fixed initiator concentration C_10_, higher C_20_ led to lower conversion due to a stronger inhibition effect. However, as shown by Figure 7, same conversion reduction was found for the same H-factor of H_0_ = [b_1_C_10_ - b_2_C_20_]. This unique feature was also predicted by Equations (13) and (20).

### 3.2. Analysis of Measured Data

Our modeling conversion profiles shown by Figure 3, Figure 4 and Figure 5 could be compared with Figure 3 of de Beer et al. [10], where to fit their data, we have adjusted the parameters of b_1_, b_2_ and rate constants (k’, k”, k_T_), but kept the same initiator concentrations (C_10_, C_20_, C_30_), as the measured data [10]. The monomer-dependence of the conversion for various resin formations is governed by the adjusted rate constants (in a relative amount), because the actual values are not available.

**Figure 8:**
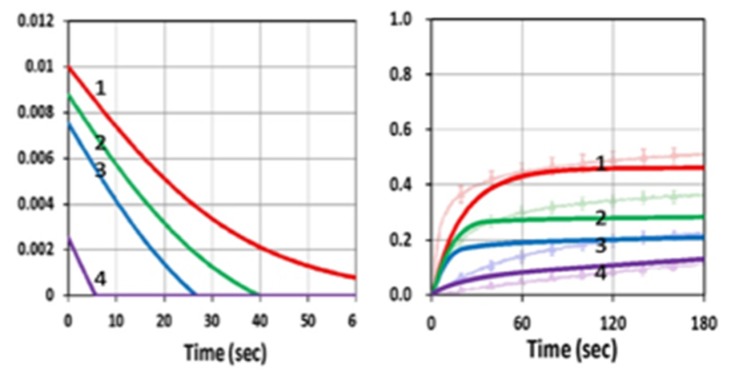
The initiation radical (left) and conversion (right) profiles for C_20_ = (0, 0.5,1.0, 3.0), for curve (red, green, blue, violet), in the presence of both blue and UV light; for b_1_ = 0.04, b_2_ = 0.002, b_3_ = 0.1 (1/s/%), k’ = 2.0 (1/s), k_48_ =10 (1/s), k_37_ = 20(1/s) and k_57_ = 0.01 (1/s). In right figure, the background is measured data from van der Laan et al. [11].

Our modeling conversion profiles shown by Figure 8 could be compared with the measured data. Figure 2 of van der Laan et al. [11]. Our modeling profiles show similar features as the measured data that inhibition effects are an increasing function of the inhibitor concentration, although our curves were slightly higher. The difference might be due to the fact that our modeling was based on idea kinetics and excluded other complex factors involved in the measurements, such as the inhibition effects due to oxygen and viscosity of the materials. Greater details of the strategies to reduce the oxygen inhibition will be discussed later (in Section 3.4). In addition, our presented conversions were for the surface layer only (with z = 0). Therefore, we expected a better fit if the spatial (z-dependence) conversion profiles were included, as shown by our Equation (15). However, most of the features and the roles of the key parameters presented based on the surface layer should also apply to a volume conversion. Spatial conversion profiles can be found in our previous study, which is limited to a single-wavelength system [13,14].

**Figure 9:**
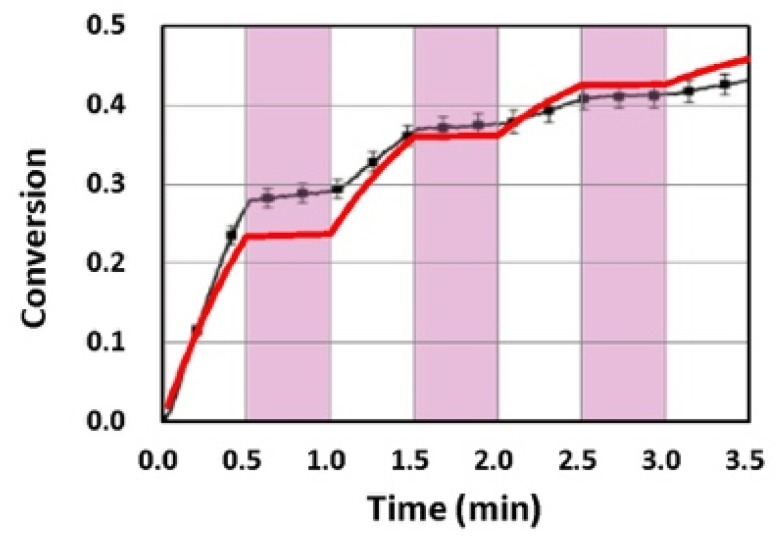
Methacrylate conversion of a bisGMA/TEGDMA resin formulated with 0.2 wt% CQ/0.5 wt% EDAB/0/5 wt% BN and subject to a continuous exposure of a blue light, but an on-off exposure of a UV-light for 0.5 min, as indicated by the violet vertical areas; where black bars are measured data from van der Laan et al. [11] and red curve is our theoretical simulation.

Van der Laan et al. [11] also reported the UV-light controlled conversion in an on-off scheme, in which methacrylate conversion of a glycidyl dimethacrylate (bisGMA) resin formulated with 0.2 wt % CQ, 0.5 wt % EDAB, a tertiary amine co-initiator and 0.5 wt % butyl nitrite (BN). Figure 9 shows the calculated conversion profile of methacrylate subject to a continuous exposure of a blue light, but an on-off exposure of a UV-light for 0.5 min, as indicated by the violet vertical areas. Measured data of van der Laan et al. [11], shown by their Figure 4, fit well to our theoretical curve (in red).

### 3.3. The General Criterion for an Efficient UV-Inhibitor

The monomer-dependence of a dual-wavelength PC was reported by van der Laan et al. [11], in which different monomers have different C=C bond rate constants (K) under the exposure of blue, UV and blue + UV. For example, bisphenol ethoxylate diacrylate (BPAEDA) resins formulated with camphorquinone (CQ) and ethyl 4-(dimethylamino)benzoate (EDAB) have a maximum conversion rate constant, for 0% butyl nitrite (BN), Kmax = 0.675 (at blue + UV), which reduces to 0.0106 (for 1%BN), a factor of 64 reduction, Therefore, it is a better candidate than trimethylolpropane triacrylate (TMPTA), which only had a three times reduction of Kmax. The above measured feature could be mathematically described by a more general criterion.

Based on our H-factor defined in Equation (14), which also governs the conversion efficacy, general criteria for efficient UV-inhibitor, or a good candidate, could be mathematically determined as follows. Defining two H factors: H_OFF_ and H_ON_ for the H-value without and with-UV, respectively, an efficient candidate (or effective UV-inhibitor) requires two conditions: (i) high enough H_OFF_ such that the conversion without UV-light larger than 50%; and (ii) high enough H_ON_ such that the conversion of both blue and UV light is reduced to lower than 20%. This concept could be further described mathematically as follows. H = H_OFF_ − H_ON_ = H_OFF_ (1 − H_ON_/H_OFF_). Therefore, a good candidate requires a large H_OFF_ and also a high H-ratio, R_H_ = H_ON_/H_OFF_. For example, for a fixed value of H_OFF_ =10, a candidate with H_ON_ = 6 leading to R_H_ = 0.6, and H = 10 × (1 – 0.6) = 40, is not as good as a candidate having a higher H_ON_ = 8 leading to R_H_ = 0.8 and H = 80), which is four times lower, presenting a stronger inhibition triggered by the UV-light.

Equation (14), H = gC_3_B_1_C_1_-B_2_C_2_, defines R_H_ = B_2_C_2_/(gC_3_B_1_C_1_). We note that B_j_ = b_j_I_j_, which is proportional to the light intensities (I_1_ for blue-light and I_2_ for UV-light) and the effective absorption constant (b_j_) governed by the quantum yield (q) and absorption coefficient at a specific wavelength. Therefore, a high H-ratio (R_H_) is determined not only by the material properties, but also the ratio of light intensity (UV/blue), and concentration ratio of the initiator and inhibitor, C_20_/C_10_. In addition, it is also rate-constant dependence, because the g-factor is given by g = 1/(k_57_ + kC_3_). Therefore, we concluded that the criterion for a good candidate is governed by collective factors, and at least by the double ratio of [I_20_C_20_.]/[I_10_C_10_]. The above criterion was an important new finding of our theoretical study, which requires further experimental study to confirm (to be discussed later).

### 3.4. Role of Oxygen and Suggested Experiments

The dual-wavelength systems presented in this study excluded the oxygen inhibition effect, which is important especially in thin polymers. There are many conventional strategies to reduce oxygen inhibition in photoinduced polymerization: working in an inert or closed environment, increasing the photoinitiator concentration, increasing the light dose or light intensity (for reduced induction time), use of multiple photoinitiators with different rate of initiation or addition of oxygen scavengers. Chemical mechanisms incorporate additives or suitably functionalized monomers, which are insensitive to oxygen, such as the thiol-ene and thiol-acrylate-Michael additive systems [17,18,19,20,21,22,23]

Recently, Childress et al. [24] reported a dual wavelength of red and UV, where red-light pre-irradiates the camphorquinone (CQ), followed by a UV excitation of the initiator, such that photosensitization and photoinitiation can be independently achieved via irradiation of the two distinct absorption bands to reduce oxygen inhibition and initiate polymerization. In their techniques, the induction time can be partially or completely eliminated (by the red light) to control (tailor) the conversion rate of UV-light initiated photopolymerization.

The theory (formulas) developed in this study could be explored further with the suggested experiments as follows:

(i) Our proposed double ratio [I_20_C_20_]/[I_10_C_10_] criterion could be experimentally justified by an experimental setup having adjustable light intensities and the initiator concentration. As shown by Figure 7, that same conversion reduction was found for the same H-factor of H_0_ = [b_1_C_10_ – b_2_C_20_]. This unique feature was also predicted by Equations (13) and (20). The experimental setup of Childress et al. [24], using various light red and UV light intensity (I_10_ and I_20_), but fixed concentrations of C_10_ and C_20_, could be easily extended for variable concentrations, such that our double ratio criterion could be justified.

(ii) A 3-wavelength (red-blue-UV) modeling system is proposed by our group (to be published elsewhere), where the red-light is used to pre-irradiate the monomer and reduce the oxygen, prior to the UV/blue light exposure as also experimentally reported by Childress et al. [24]. This 3-wavelength strategy could further enhance the conversion efficacy (by reduced oxygen), and also the photopolymerization confinement effects.

(iii) Microfabrication and utilized to reduce the deposition steps and to obtain a monolithic product was reported by Alvankarian et al. [25], Chen et al. [26] and Wu et al. [27], in which structures of arrays of pillars in photo-cross-linkable films were measured by irradiation with a periodic array of microscale optical beams under ambient conditions. Curing depth and inhibition zone control are the key factors for microfabrication, in which the influencing factors of curing depth include: light intensity and exposure time (or dose), the initial concentration of initiators and oxygen and the external supply of oxygen. The optical beams experience a self-focusing nonlinearity owing to the photopolymerization-induced changes in refractive index, thereby concentrating light and driving the concurrent, parallel growth of microscale pillars along their path length [27]. In the microfabrication system, formation of radical decreases over depth due to the reduction of light intensity and initiator concentration, and increase in oxygen inhibition. Under ambient conditions, oxygen diffuses into the film and consumes radicals. The balance of radical production and oxygen inhibition gives rise to the inhibition zone, where the polymerization is completely suppressed. The 3-wavelength technique proposed in (ii) could be used to tailor the inhibition zone by controlled oxygen inhibition and radical production. Our formulas given by Equations (25) and (29) provide influencing factors of the inhibition zone and curing depth.

(iv) As reported by van der Laan et al. [11], the dual-wavelength confinement was achieved by inhibition volume depth controlled by varying the ratio of the intensities of the UV and blue lights, thus surface topographical patterning can be achieved in a single exposure/layer with no stage translation. Therefore, our dual-wavelength system and the extended 3-wavelength system could offer potential new applications for various integrated designs for 3D printed tissue scaffolds as reviewed by Egan [28]. Our developed formulas could be also useful to analyze the microscale features and mechanical efficiency [29], which are closely related to the curing depth and efficacy given by Equations (20) and (29).

(v) Differential photocalorimetry (DPC) devices such as DuPont 2100-Thermal-Analyzer provide a powerful analytical tool for the characterization of UV curable materials [30,31,32]. Recent studies in the advancement of photopolymerization technology have explored combinations of free radical and cationic initiated materials, or hybrid photopolymers (HP), which can provide unique product performance unattainable with the individual photopolymers. The various types of free radical acrylate, cationic epoxy and vinyl ether and hybrid photopolymers were differentiated by their characteristic DPC results [29]. For examples, acrylate homopolymers were characterized by lack of post UV exposure thermal cure and by their distinct and highest glass transition temperatures; diepoxy homopolymer and HP were characterized by their thermal post UV exposure curing behavior and divinyl ether homopolymer and HP were characterized by fast photocure speeds and by the color change of the UV cured sample to red after heating to high temperature [30]. Our calculated conversions shown by Figure 3, Figure 4 and Figure 5 were consistent with the measured data of de Beer et al. [10] and could be further analyzed and compared with that of DPC, which, however, were not within the scope of this study.

## 4. Conclusions

We theoretically demonstrated that without the UV-light, for a given blue-light intensity, higher initiator concentration (C_10_) and rate constant (k’) led to higher conversion, governed by a scaling law of k[gB_1_C_1_]^0.5^. However, the coupling factor b_1_ played a different role that higher b_1_ led to higher conversion only in the transient regime; whereas higher b_1_ led to lower steady-state conversion. For a fixed initiator concentration C_10_, higher C_20_ led to lower conversion due to a stronger inhibition effect. However, the same conversion reduction was found for the same H_0_ = [b_1_C_10_ – b_2_C_20_]. Conversion of blue-only was much higher than that of UV-only and UV-blue combined, such that the UV-light served as the turn-off (trigger) mechanism for the purpose of PC. The UV-light initiated inhibition effect was strongly monomer-dependent and different monomers had different C=C bond rate constants and conversion efficacy.
